# Comprehensive characterization and building of National Registry of von Hippel–Lindau disease in Brazil

**DOI:** 10.1002/mgg3.2136

**Published:** 2023-01-10

**Authors:** Tabatha Nakakogue Dallagnol, Eduardo Da Cás, Odery Ramos Junior, José Cláudio Casali‐da‐Rocha

**Affiliations:** ^1^ Department of Medical Oncology Hospital Erasto Gaertner Curitiba Brazil; ^2^ Faculty of Medicine Universidade Positivo Curitiba Brazil; ^3^ Department of Internal Medicine Hospital de Clínicas – Universidade Federal do Paraná Curitiba Brazil; ^4^ Department of Oncogenetics Hospital do Câncer A.C. Camargo São Paulo Brazil

**Keywords:** clinical database, follow‐up, genotype–phenotype correlation, VHL, von Hippel–Lindau disease

## Abstract

**Background:**

Von Hippel‐Lindau (VHL) disease is an autosomal dominant disorder caused by pathogenic variants in VHL gene. The common manifestations include hemangioblastomas (HB) of the central nervous system (CNS) and retina (RH); pheochromocytoma (PHEO); clear cell renal cell carcinoma (ccRCC); pancreatic and renal cysts (PRC) and pancreatic neuroendocrine neoplasm (PNEN).

**Methods:**

The first characterization of VHL in Brazil was published in 2003 and included 20 families with a history of VHL. The aim of this study was to expand the previous Brazilian cohort to include more families, as well as to collect prospectively both clinical and molecular characteristics of patients with VHL to build the VHL Brazilian Registry (VHLBR). Patients with VHL were selected through review of data from medical records of experts and from social networks of support for families with VHL in Brazil.

**Results:**

A total of 142 subjects representing 62 unrelated Brazilian families with VHL were registered. The mean age of VHL onset was 28.78 years old and 128 individuals (90.1%) had at least one VHL‐related lesion. CNS HB was the most common manifestation occurring in 91 (71%) patients, followed by multiple PRC (48.4%), RH (39.8%), ccRCC (28.9%), PHEO (12.5%) and PNEN (7.8%). Of the 97 subjects whose presence of VHL variants was confirmed, 51 (52.6%) had missense variants, 22 (22.7%) large deletions, 10 (10.3%) frameshift, 7 (7.2%) splice site, 4 (4.1%) nonsense and 3 (3.1%) in‐frame deletions. Regarding surveillance, 115 (81%) participants had at least one physician responsible for their outpatient follow‐up; however, 69 (60%) of them did not report a regular frequency of tests.

**Conclusion:**

We built the largest prospective VHLBR with organized collections of clinical and genetic data from families with VHL, which will be helpful to guide policies for VHL care and oncogenetics in Brazil. Although there have been improvements in diagnosis and clinical screening methods, VHL care in Brazil is still deficient, especially regarding surveillance and regular medical appointments with experts.

## Graphical abstract

Von Hippel‐Lindau (VHL) is a hereditary syndrome that affects several visceral organs and the central nervous system. In this study, we report clinical and genetic characteristics of 142 participants with VHL. Therefore, we created the largest Brazilian cohort of VHL patients to define the main clinical characteristics and follow‐up of VHL disease, as well as to investigate genotype‐phenotype correlations.

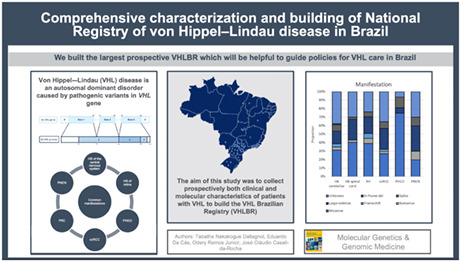

## 1 BACKGROUND

Von Hippel–Lindau (VHL) disease (OMIM #193300) is an autosomal dominant disorder caused by germline *VHL* gene mutations that affects 1:36,000 individuals worldwide and has similar prevalence in both genders (Lonser et al., 2003; van Leeuwaarde et al., 2018). This disease predisposes individuals to develop hemangioblastomas (HB) of central nervous system (CNS), hemangioblastomas of retina (RH), clear cell renal cell carcinomas (ccRCC), pheochromocytoma (PHEO), pancreatic neuroendocrine neoplasms (PNEN), endolymphatic sac tumors (ELST), epididymal or broad ligament cystadenomas and cystic disease in several organs, including kidneys, pancreas, and liver (Maher et al., 2011; Nordstrom‐O'Brien et al., 2010). The penetrance of VHL is age‐related and almost complete by 60–75 years old (Binderup et al., 2017; Poulsen et al., 2010).

The tumor suppressor gene of VHL is located on chromosome 3p25 and consists of only three exons (Latif et al., 1993; Ong et al., 2007). This gene encodes the VHL protein (pVHL) that seems to have multiple functions in regulating proteolytic degradation of the alpha subunits of the transcription factors of hypoxia‐inducible factor‐1 (HIF‐1) and hypoxia‐inducible factor‐2 (HIF‐2) (Chittiboina & Lonser, 2015; Ong et al., 2007). The HIF transcription factors have a critical role in orchestrating cellular responses to hypoxia and in regulating transcription of a wide variety of genes related to angiogenesis, proliferation, and metabolism (Gläsker et al., 2020; Maxwell et al., 1997).

Clinically, VHL disease is categorized into VHL type 1 or type 2 according to the familial phenotype. Type 1 regards those with typical VHL manifestations, such as CNS HB, ccRCC, and RH, but does not include PHEO. Therefore, in the presence of PHEO, it is categorized as type 2, which is subdivided into: 2A – PHEO and other typical VHL manifestations except ccRCC; 2B – full spectrum of VHL disease including PHEO, ccRCC, and other manifestations; 2C – isolated PHEO (Kim & Kaelin, 2004; Maher et al., 1996; Neumann & Wiestler, 1991).

Due to its lifetime multi‐organ involvement and phenotypic heterogeneity, the clinical management, treatment, and counseling of this population are challenging. Furthermore, the nature of VHL requires patients to be prospectively screened for new manifestations, demanding a skilled multidisciplinary team, as well as a compliant patient (Nordstrom‐O'Brien et al., 2010).

Surveillance is crucial not only for detecting new lesions at an early stage, but also for monitoring small asymptomatic lesions (Priesemann et al., 2006). Without clinical surveillance, the median life expectancy of patients with VHL is 41 to 49 years old (Poulsen et al., 2010). The most frequent causes of VHL‐related death are neurological damage due to CNS lesions and metastasis of ccRCC (Lonser et al., 2003).

The available data of the Brazilian population correlating phenotype and genotype of the VHL is limited. The first characterization of VHL in Brazil was published in 2003 and included 20 families with VHL (Rocha et al., 2003). The project lasted 12 years and provided access to genetic testing for 85 participants, from 1998 to 2010 (data not published). After 2010, 42 new families were identified, but *VHL* testing became available only in private laboratories for those with health insurance, while for the patients that depended on the Brazilian public health system, the testing was not available (Ashton‐Prolla & Seuanez, 2016). The aim of this study was to expand the previous Brazilian cohort in order to include more families, as well as to collect prospectively both clinical and molecular characteristics of families with VHL, to assess their access to *VHL* testing, their follow‐up, their treatment modalities, and their survival. Furthermore, another aim was to build the von Hippel–Lindau Brazilian Registry (VHLBR).

## 2 MATERIALS AND METHODS

### 2.1 Database

The national database gathers information on demographics, family history, VHL‐related manifestations (onset, location, type, pathology, treatments), access to genetic testing, genetic variant, screening plan, and modalities.

Mutation analyses of the *VHL* gene included a variety of methods, depending on the availability of genetic techniques at the time of testing. All variants are classified according to the American College of Medical Genetics and Genomics (ACMG). Furthermore, databases and resources such as ClinVar, CIVic, and VarSome were consulted.

### 2.2 Patients

Patients with VHL were retrospectively selected through review of experts' medical records from ophthalmology, urology, endocrinology, oncology, genetics, and neurosurgery healthcare services. In addition, the families were also selected from social networks supporting people with VHL in Brazil.

Patients with a clinical diagnosis of VHL regardless of confirmatory genetic testing, as well as those with a positive VHL family history and confirmed genetic variant, were included in the analysis. Demographic, genetic, and clinical data were collected and included in the database. The participants were prospectively monitored regarding clinical manifestations of the syndrome, treatments, and surveillance.

Previously, 20 families (family VHL 1 to 20) had already been studied and reported, but we proposed to update the clinical data of these families, as well as data regarding their follow‐up and treatments. The inclusion of the other 42 families (family VHL 21 to 62) is original in this study. The follow‐up period was up to 22 years (the first 20 families were included in 1998) and the data inclusion in the database will be continuous from now on.

### 2.3 Analysis

Data obtained from the 142 participants were double‐entered into Microsoft Excel® spreadsheets and validated for data errors. The analysis was conducted after codification of data and exportation to the software SPSS version 2.0. Descriptive statistics, such as frequencies and percentages, were used to describe data.

## 3 RESULTS

**FIGURE 1:**
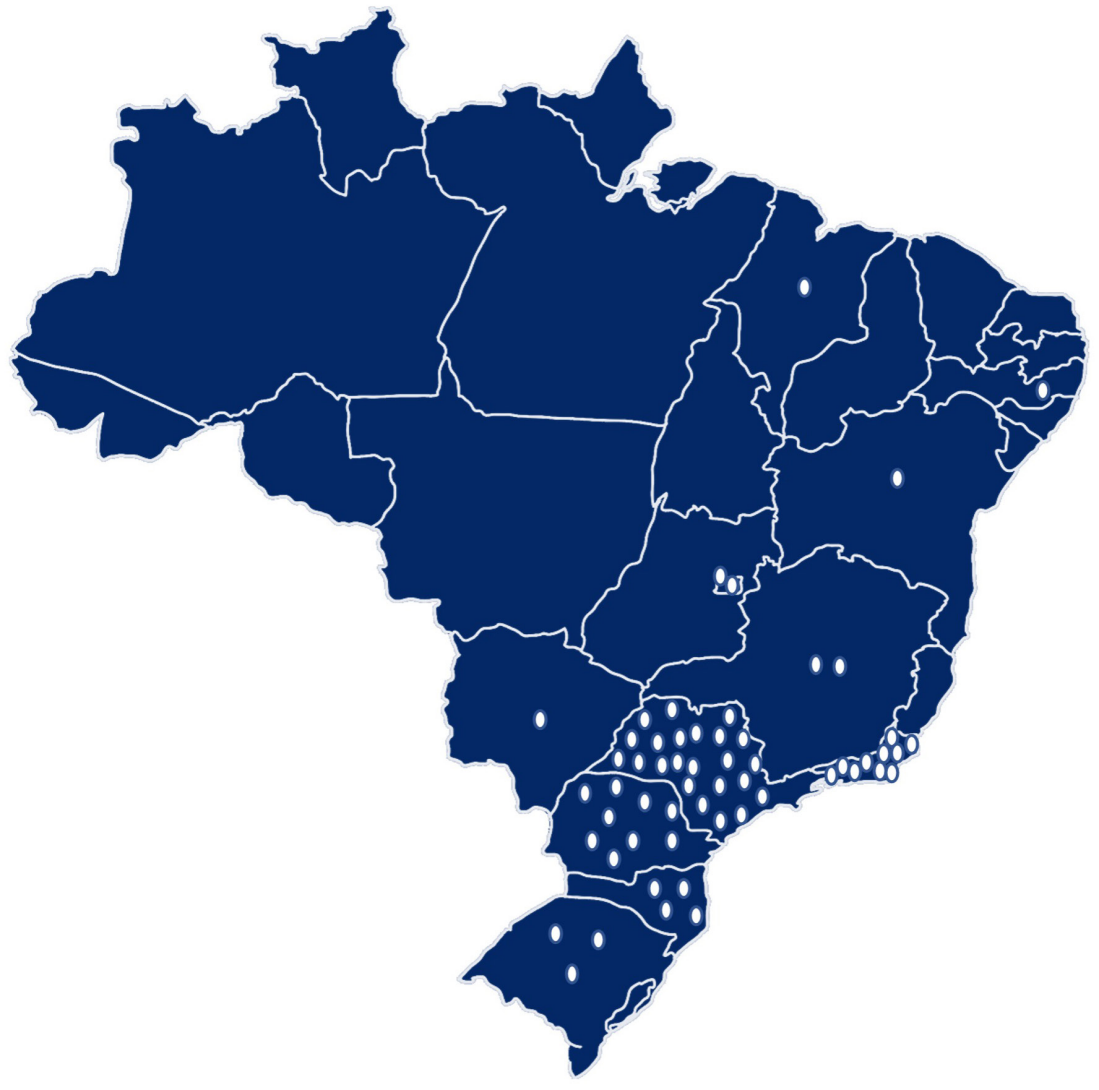
Brazilian georeferencing of VHL families (each dot represents 1 family with VHL).

A total of 142 participants representing 62 unrelated families with VHL were included. Their ages ranged from 11 to 83 years old and they had a mean and median age of 43 and 42.5 years old, respectively. There was a slightly higher proportion of females (53.5%) in comparison to males (46.5%) (Table 1). We observed that 35 families (56.5%) were from the Southeast, 16 (25.8%) were from the South, 4 (6.5%) were from the Central‐West and 4 (6.5%) were from the Northeast Brazilian region. Moreover, three families are currently living abroad (in Ecuador, Uruguay, and Portugal) (Figure 1).

**TABLE 1:** Demographic information of participants

	*N* (range/%)
Total number of participants	142
Total number of families	62
Median age	42.5 (11–83)
Female	76 (53.5)
Male	66 (46.5)
Probands^a^	62 (43.7)
De novo VHL^b^	16 (11.2)
Genetic testing	109 (76%)
Identified *VHL* variant	97 (89%^d^)
Unknown testing results^c^	12 (11%)
No genetic testing	34 (24%)
Participants with clinical manifestations	128 (90.1%)
Asymptomatic carriers	14 (9.9%)

^a^ Proband is defined as an affected VHL patient due to a symptomatic VHL manifestation.

^b^ *De novo* VHL is suspected in the absence of prior family history of clinical VHL.

^c^ Genetic testing was performed and the results are unknown or awaiting the report.

^d^ Percentage of those with genetic testing.

The Brazilian Unified National Health System (SUS) was the care provider for more than half of the families (53.2%), while in 29 families (46.8%) at least 1 member had access to health insurance.

Of all the 142 VHL cases, 109 underwent genetic. Ninety‐seven participants had an identified variant in the *VHL* locus; ten subjects had a record of genetic testing but with no descriptive results and two participants are awaiting the report. Among those participants that were tested, 78% accessed genetic testing through previous research protocol and 22% through health insurance or private coverage.

*De novo* cases of VHL were suspected in 16 individuals who had no prior family history of the disease. Of the 97 participants with confirmation of *VHL* variants, 51 (52.6%) had missense variants, 22 (22.7%) had large deletions, 10 (10.3%) had frameshift, 7 (7.2%) had splice site, 4 (4.1%) had nonsense and 3 (3.1%) had in‐frame deletion.

**FIGURE 2:**
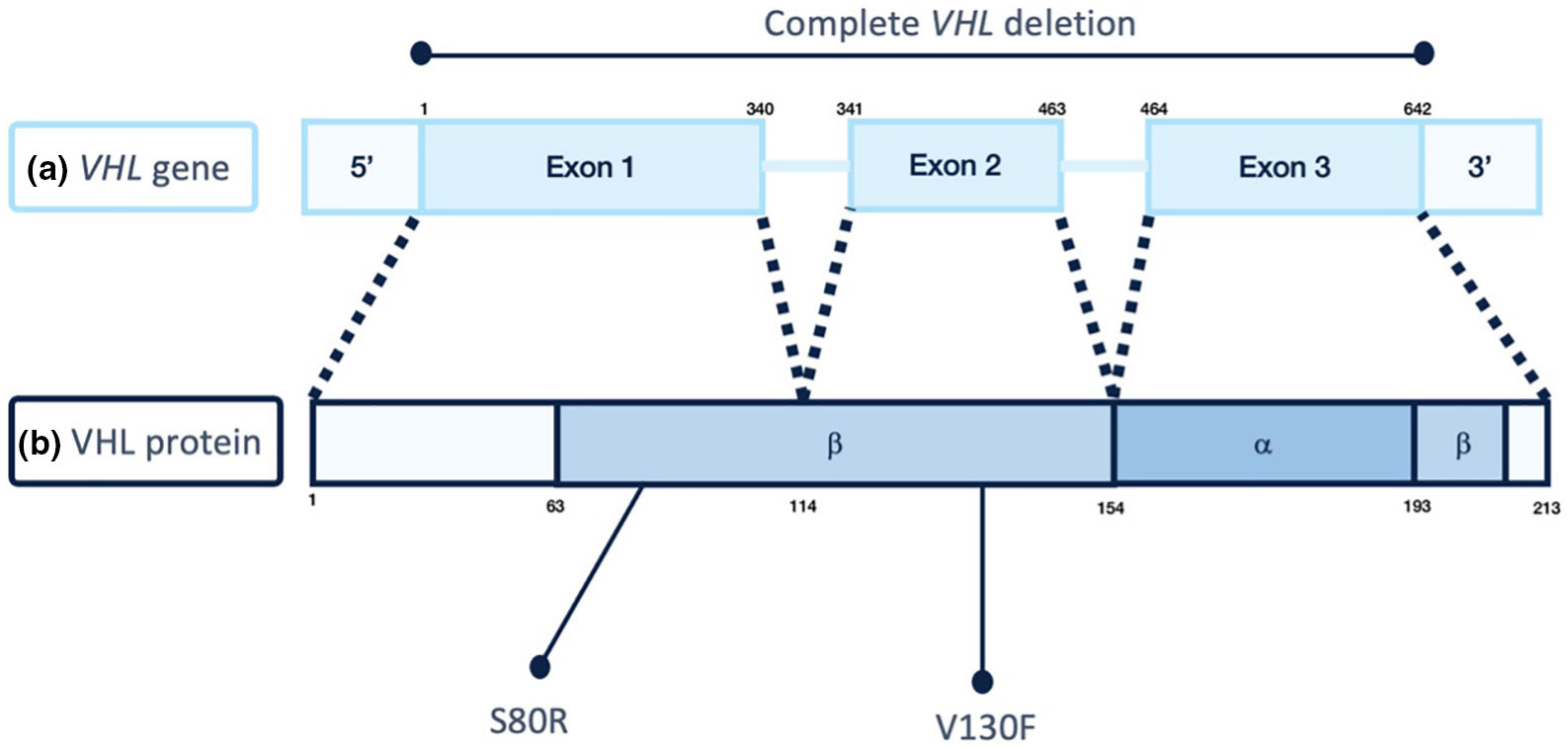
von Hippel–Lindau gene and protein structure. (a) The *VHL* gene comprises three exons. Nucleotide number is indicated above the gene structure as well as the representation of complete *VHL* deletion. (b) The VHL protein structure with α‐domain and β‐domain. Codon number is indicated below the protein structure. The two protein changes due to the most common missense variants, in our cohort, are indicated below the figure. Amino acids are in single‐letter notation.

The most common variants identified were NM_000551.4(VHL) c.238A > C (p.Ser80Arg) in eight individuals, belonging to the same pedigree, and *VHL* complete deletion in ten individuals, belonging to three distinct pedigrees. The most common variant among independent families was NM_000551.4(VHL) c.388G > T (p.Val130Phe), present in four different families (Figure 2; Table S1).

VHL‐related clinical lesions were present in 128 individuals (90.1%) and 14 (9.9%) were asymptomatic carriers. Of those with clinical manifestations, 113 (80%) had leastwise one supporting exam (radiological or pathological correlation) and 15 (11%) were self or medical assistant reported. The majority of participants had at least two different manifestations, demonstrating the overlap of different manifestations in single individuals.

The mean age of VHL onset, defined as the first diagnosis of any VHL‐related manifestation detected symptomatically or asymptomatically through VHL surveillance, was 28.78 years old, with a median of 26 years old. The age of onset did not differ significantly between VHL type 1 or 2. Cerebellar HB was the most frequently reported manifestation in this cohort (30.5%), followed by RH (16.4%). In families with VHL type 2 (30 families), PHEO was the initial manifestation for 6 participants (20%).

The most prevalent manifestation in our cohort was CNS HB, occurring in 91 (71%) patients, with cerebellar (80 individuals) and spinal cord (39 individuals) HB being the most common subtypes. RH was identified in 51 participants, with a mean onset age of 25 years old. The ccRCC was present in 37 participants (28.9%) and renal cysts in 62 (48.4%), with mean onset age of 40 and 37 years old, respectively. We found PNEN in ten participants (7.8%) and 62 (48.4%) had pancreatic cysts, which were more common and had an earlier age of onset in comparison to PNEN (34.1 years old and 40 years old, respectively). We also identified PHEO in 16 participants (12.5%), with a mean onset age of 28.1 years old, and we observed two ELST diagnoses in this cohort, with a mean onset age of 27.5 years old. Epididymal cysts and cystadenomas were found in 15 participants (23% of male participants with VHL), with a mean onset age of 24.8 years old. In this cohort, there were no diagnoses of broad ligament cystadenoma (Table 2).

**TABLE 2:** Prevalence of manifestations and onset age

Clinical manifestation	Prevalence (%)	Mean onset age (years old)
HB cerebellar	80 (62.5%)	28.8
HB spinal cord	39 (30.5%)	31
HB brainstem/supratentorial	18 (14.1%)	29.1
HB retinal	51 (39.8%)	25.6
Clear cell renal cell carcinoma	37 (28.9%)	40
Renal cysts	62 (48.4%)	37
Pheochromocytoma	16 (12.5%)	28.1
Pancreatic neuroendocrine neoplasm	10 (7.8%)	40
Pancreatic cysts	62 (48.4%)	34.1
Epididymal cysts/cystadenomas	15 (23%^a^)	24.8
Endolymphatic sac tumors	2 (1.6%)	27.5

Abbreviation: HB, hemangioblastoma.

^a^ Percentage of male participants with VHL.

**FIGURE 3:**
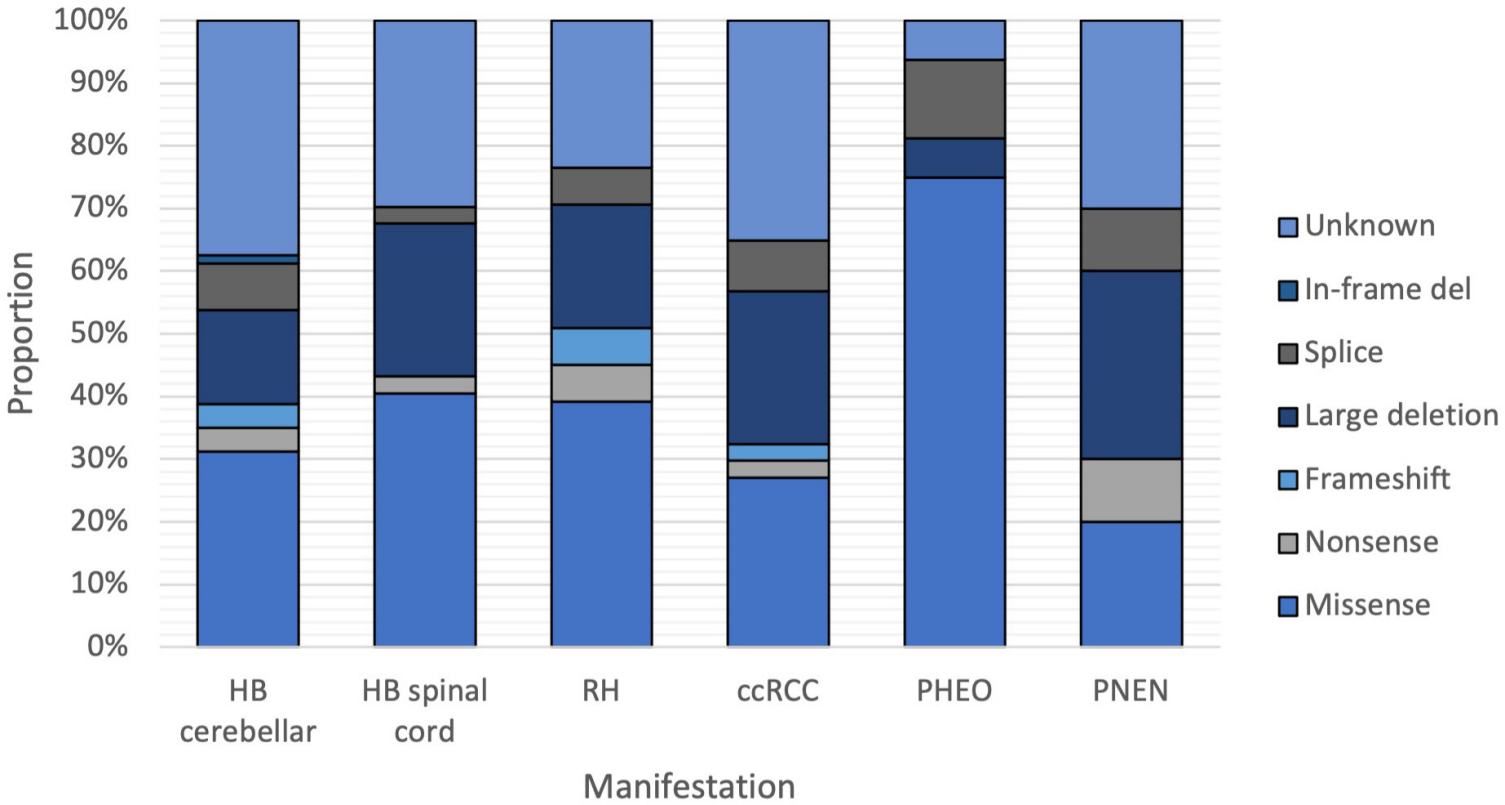
Proportion of variant types by main VHL‐related manifestation. ccRCC, clear cell renal cell carcinoma; HB, hemangioblastoma; PHEO, pheochromocytoma; PNEN, pancreatic neuroendocrine neoplasm; RH, retinal hemangioblastoma.

According to the familial phenotype, families were categorized in: (a) VHL type 1, 50 families (81%); (b) VHL type 2A, 7 families (11%); (c) VHL type 2B, 5 families (8%); and none were VHL type 2C (Figure S1). To identify preliminarily genotype–phenotype correlations, we investigated the relationship between variant types and main VHL‐related manifestations (Figure 3). Of the families with VHL type 1, 49.3% had missense, 19.2% had large deletions, 13.7% had frameshift, 8.2% had splice site, 5.5% had nonsense, and 4.1% had in‐frame deletion. As expected, VHL type 2 families mainly had missense mutations (62.5%).

Regarding the treatments that participants with clinical manifestations had already undergone, 22.7% had been subjected to multiple modalities, with surgery (75.8%) and minimally invasive procedures, such as radioablation/embolization (18.8%), being the most frequent (Figure S2).

During the follow‐up time (up to 22 years), 39 participants (27.5%) died. The median age of death was 43 years old (ages ranging from 22 to 73 years old) and the most frequent causes of VHL‐related death were postoperative complications related to CNS HB interventions (38.5%) and the systemic progression of ccRCC (25.6%) with metastasis.

In this cohort, the medical specialties that were mainly responsible for the longitudinal care of the families were genetics (39%) and neurology/neurosurgery (22%); however, 19% of participants did not report to have one physician responsible for their outpatient care (Figure S3). Among those participants in medical follow‐up, 69 (60%) did not report undergoing imaging or laboratory tests in general with a regular frequency (Figure S4). Moreover, among those participants who have known VHL‐related target lesions (91 patients), 65.9% did not undergo regular monitoring either (Figure S5).

## 4 CONCLUSION

Von Hippel–Lindau is a hereditary multisystem syndrome that has peculiar manifestations and affects several visceral organs and the CNS. In this study, we report clinical and genetic characteristics of 142 participants with VHL. Therefore, we created the largest Brazilian cohort of VHL patients to define the main clinical characteristics and follow‐up of VHL disease, as well as to investigate genotype–phenotype correlations. The families with VHL included in our study are potentially representative of the Brazilian population since they came from different regions of the country.

For prevalence analyses, we used the international diagnostic VHL criteria for direct comparisons with previous estimates (Maddock et al., 1996; Maher et al., 1991) (Table 3). In our study, the overall frequency of cerebellar HB was similar to the findings reported in 1990 by Maher et al. and in 2007 by Ong et al. (Maher et al., 1991; Ong et al., 2007). The prevalence of RH in our study was 39.8% (51 of 142). There is plenty of variability in the frequency reported in the literature according to heterogeneity and diagnostic methods. Maher et al., Ong et al., and Wong et al. found an RH frequency of 59%, 73% e 27.9%, respectively (Maher et al., 1991; Ong et al., 2007; Wong et al., 2016).

**TABLE 3:** Comparison of frequency of main clinical manifestations of VHL syndrome across different cohorts

Clinical manifestation	Our study (*n* = 142)	Wong et al. (2016) (*n* = 154)	Maher et al. (1991) (*n* = 573)	Ong et al. (2007) (*n* = 152)
HB cerebellar	62.5%	–	57%	59%
HB spinal cord	30.5%	–	25%	13%
HB CNS	71.1%	81.2%	–	–
RH	39.8%	27.9%	73%	59%
ccRCC	28.9%	57.8%	35%	28%
Pheochromocytoma	12.5%	14.9%	20%	7%

Abbreviations: ccRCC, clear cell renal cell carcinoma; CNS, central nervous system; HB, hemangioblastoma; RH, retinal hemangioblastoma.

The mean age of ccRCC diagnosis among our patients with VHL was 40 years old, which is similar to other studies with VHL population and can be considered early when compared to sporadic ccRCC cases (Binderup et al., 2017). The overall frequency of ccRCC in this cohort was 28.9%, similar to that reported by Maher et al. but lower than that reported by both Ong et al. and Wong et al. (Maher et al., 1991; Ong et al., 2007; Wong et al., 2016). A possible reason for this difference is the criteria used to define ccRCC, since it is currently possible to consider radiological diagnosis without histological proof; however, in our cohort, we depended on the information provided by the patients and the referral of follow‐up images, and as aforementioned, patients did not undergo regular examinations as was expected.

We found 12.5% of PHEO, which is in agreement with recent publications (Ong et al., 2007; Wong et al., 2016). The discrepancy when compared to the Maher's cohort could be explained not only by improvements in screening and detection of PHEO, but also by increased incidence based in age‐dependent penetrance (Maher et al., 1991).

Age of onset of main VHL clinical manifestations in our patients is similar to the previously published data (Feletti et al., 2016; Maher et al., 1991; Ong et al., 2007; Salama et al., 2019; Varshney et al., 2017; Wong et al., 2016). In our cohort, 9.9% (14 of 142 participants) were VHL variant carriers without manifestations.

Estimates of de novo VHL cases have been reported ranging from 3% to 21% (Nordstrom‐O'Brien et al., 2010; Richards et al., 1995). In our study, we estimate that the disease was caused by de novo mutations in 11.2% (16 of 142) cases. However, our assessment was based on family history and was genetically confirmed in only four families. In the other families, proband's parents had not been genetically tested and could represent asymptomatic or mildly affected or germline mosaicism (Sgambati et al., 2000). In particular, two cases had a lack of access to genetic‐relative family health history, one due to early death of parents and separation from other relatives, and the other was an adoptee proband with separation from biological parents.

The spectrum of known mutations in the *VHL* gene is wide and the frequency of variant types that we found was similar to cohorts published previously. In our study, missense mutations in the *VHL* gene were the most common mutation, a fact that is in agreement with findings presented by Nordstrom‐O'Brien et al. and Wong et al. (Table 4), which comprised different ethnic populations (Nordstrom‐O'Brien et al., 2010; Wong et al., 2016).

**TABLE 4:** Comparison of the distribution of genetic mutations in the von Hippel–Lindau (*VHL*) gene across different cohorts (east Asian and Western patients)

Type of VHL mutation	Our study (%)	Wong et al. (2016) (%)	Nordstrom‐O'Brien et al. (2010) (%)
Missense	52.6	40.9	52.1
Frameshift	10.3	8.4	13.3
Nonsense	4.2	11.7	11.3
Large deletion	21.6	32.5	10.8
Splice	8.2	4.5	6.8
In‐frame deletion	3.1	1.3	5.6

We preliminary examined genotype–phenotype correlation in VHL families and our data support the previously distinction described in the literature between VHL types 1 and 2 regarding missense variants and their association with a higher risk of pheochromocytoma (Crossey et al., 1994; Maher et al., 1991; Zbar et al., 1996); 80% of our tested individuals with PHEO presented a missense variant. In our study, 39.2% of tested patients with RH had missense variants, followed by 19.6% with large deletions. This is similar to prior findings published by Mettu et al. and Wong et al. (Mettu et al., 2010; Wong et al., 2016). Our analysis of pooled information also showed that nonsense and frameshift mutations occur just in VHL type 1, which had also been discussed in previous publications (Nordstrom‐O'Brien et al., 2010).

Regarding access to genetic tests, there is no financial support in SUS for these tests and only recently the coverage of genetic testing by private healthcare insurance became mandatory in Brazil. This feature is reflected in our data, which shows that 78% of participants had access to genetic testing only by means of the previous research protocol. Major challenges still lay ahead in order to facilitate access for individuals who use the public healthcare system to better genetic care.

According to the literature, prophylactic surveillance could reduce VHL‐associated morbidity and mortality (Poulsen et al., 2010). Multiple guidelines help to systematize the evaluation of VHL according to age‐dependent methods and preferably noninvasive techniques, such as those proposed by the US National Institutes of Health, the Danish VHL Coordination Group, and VHL Alliance. In general, these guidelines entail complete clinical examination, including neurological examination, ophthalmological evaluation with fundoscopy, audiometry, imaging of CNS, kidney, pancreas, and liver, as well as laboratory tests (Lonser et al., 2003; Mourão et al., 2022; Poulsen et al., 2010; VHL Alliance, 2020).

Regarding imaging, the vast majority of international recommendations indicate the use of CNS and abdominal magnetic resonance (MR) considering several advantages: no radiation exposure, more sensibility to evaluating smaller lesions and potential renal impairment, or allergy that limits iodinated intravenous.

contrast agents (Ganeshan et al., 2018). However, there are discussions about cost and accessibility as well as repeated use of paramagnetic contrast. The prevalence of gadolinium accumulation in the dentate nuclei and pale globes increased linearly according to the number of contrast injections that patients with VHL were subjected to, especially after more than 5 serial examinations (Vergauwen et al., 2018). In this scenario, new surveillance approaches emerge, such as whole‐body MR protocol – potentially a more effective and safer strategy (Vanbinst et al., 2019).

Modifications of surveillance schedules could be done by physicians familiar with patients and their family history. Therefore, an adapted follow‐up plan based on the patient's manifestations and circumstances should take place to balance early manifestation detection and over investigation, not only due to patients' psychological strain, but also due to the financial impact on healthcare systems (VHL Alliance, 2020).

In our cohort, VHL surveillance was evaluated according to the frequency of medical appointments, indication of a physician responsible for their outpatient follow‐up, and their examination routine. Our findings suggest an important gap in regular clinical follow‐up of families with VHL, both in SUS and in private health care. Since VHL care is complex, this emphasizes the need to build a clear and feasible clinical‐radiological screening program for VHL‐related lesions adapted for multiple Brazilian realities as proposed in Table 5.

**TABLE 5:** General proposal for a Brazilian protocol of VHL monitoring

Target lesion related VHL	Type of surveillance	Age of surveillance initiation	Time intervals between examinations	Comments
	History and physical examination (neurologic examination, auditory and vestibuloneural testing, visual and catecholamine excess symptom assessment)	1 year	12 months	Due potential difficulties in accessing several specialists in the public health service, proposal for centralized FUP by a trained MDT
Pheochromocytoma	Blood pressure and pulse	2 years	12 months	Encourage monitoring close to home to increase adherence
Retinal hemangioblastoma	Ophthalmic examination	Before 1 year	12 months	Maintenance of this frequency up to at least patient's thirties considering mean onset age
Hemangioblastomas	Imaging of the CNS (brain and neuroaxis ‐ MRI)^a^	15 years	24 months	One‐day radiological approach to facilitate compliance and access
Clear cell renal cell carcinoma Pheochromocytoma Pancreatic neuroendocrine neoplasm	Imaging of the abdomen (MRI)^a^	15 years	24 months	One‐day radiological approach to facilitate compliance and access
Pheochromocytoma	Plasma metanephrines	5 years	12 months	
Endolymphatic sac tumors	Audiometry	11 years	24 months	
Endolymphatic sac tumors	MRI internal auditory canal^a^	15 years	One baseline examination	Due to low prevalence, conditioned to clinical evaluation/audiometry

Abbreviations: FUP, follow‐up; MDT, multidisciplinary team; MRI, magnetic resonance image.

^a^ Issues related to healthcare resource allocation could affect access to MRI in all regions of Brazil, therefore the following imaging priority order may be applied (aware of inherent limitations, especially to neuroaxis): MRI (with and without contrast) > MRI (without contrast) > CT (with contrast) > CT (without contrast) > US (kidneys, adrenals, and pancreas only).

We demonstrate the potential utility of a comprehensive VHL database, encompassing the standardized collection of clinical and genetic data. Ultimately, we expect that the national database will provide the basis for a more targeted VHL surveillance and counseling, with the possibility of broad collaboration, definition of specific risks in the Brazilian population, and a better understanding of VHL.

## 5 LIMITATIONS

This study had some limitations. The inclusion of patients with VHL from referral by experts and from social networks for supporting patients and families with VHL may contribute to a selection bias. Moreover, given the low frequency of regular follow‐up by patients with VHL, some clinical manifestations may have been missed if they were not properly screened; thus, it may lead to underestimation of the total number of affected individuals. When a participant did not report having a particular manifestation, in some cases we could not determine if this is due to negative results of investigations or if the patient was asymptomatic and, thus, did not undergo investigation. Furthermore, considering that we had families from multiple Brazilian regions, almost all of our interviews were carried out online or based on answers provided by forms filled out by patients. Also, we depended on the referral of the examinations that the patients had already undergone in order to analyze possible abnormal results regardless of patients' symptoms. These aspects limited our access to data in cases of low engagement in our study's demands.

## AUTHOR CONTRIBUTIONS

Conceptualization of study: Tabatha Nakakogue Dallagnol, Eduardo da Cas, Odery Ramos Junior and Jose Claudio Casali‐da‐Rocha; Data acquisition: Tabatha Nakakogue Dallagnol and Eduardo da Cas; Data analysis and manuscript preparation: Tabatha Nakakogue Dallagnol, Odery Ramos Junior and Jose Claudio Casali‐da‐Rocha. All authors approved the final article.

## CONFLICT OF INTEREST

No conflicts of interest.

## ETHICS STATEMENT

This study has been approved by the Human Research Ethics Committee of the institution (N° 12891719.6.0000.0098). All participants signed an Informed Consent Form. Genetic counseling was offered to all participants, and they were advised about the VHL clinical screening program they should participate in throughout their lifetime.

## Supporting information


Appendix S1

## Data Availability

The data that support the findings of this study are available on request from the corresponding author (TND). The data are not publicly available due to privacy or ethical restrictions.
