# Supplementary material for: Comprehensive characterization and building of National Registry of von Hippel–Lindau disease in Brazil
Source: Mol Genet Genomic Med. 2023 Jan 10;11(4):e2136. doi: 10.1002/mgg3.2136 (PMC10094063; doi:10.1002/mgg3.2136)

**Supplementary material**

Table S1 – VHL genetic and manifestation information of participants.

| Subject | cDNA (NM_000551.4) | Nt | Protein Change | Codon | Variant Type | CNS | ccRCC | PNEN | RH | PPGL | Epi |
| --- | --- | --- | --- | --- | --- | --- | --- | --- | --- | --- | --- |
| 1 | c.224_226delTCT | 224 | p.Phe76del | 76 | In-frame del | N | N | N | N | N | N |
| 2 | Partial deletion | NA | NA | NA | Large deletion | Y | Y | N | N | N | N |
| 3 | Complete Deletion | NA | NA | NA | Large deletion | N | Y | N | N | N | N |
| 4 | c.407T>C | 407 | p.Phe136Ser | 136 | Missense | Y | Y | N | N | N | N |
| 5 | c.407T>C | 407 | p.Phe136Ser | 136 | Missense | Y | Y | N | N | N | N |
| 6 | c.407T>C | 407 | p.Phe136Ser | 136 | Missense | Y | N | N | Y | N | N |
| 7 | c.407T>C | 407 | p.Phe136Ser | 136 | Missense | N | N | N | N | N | N |
| 8 | c.407T>C | 407 | p.Phe136Ser | 136 | Missense | Y | N | N | N | N | N |
| 9 | c.407T>C | 407 | p.Phe136Ser | 136 | Missense | Y | N | N | N | N | N |
| 10 | c.262T>C | 262 | p.Trp88Arg | 88 | Missense | N | N | N | Y | N | N |
| 11 | c.305delC | 305 | p.Pro102ArgfsTer? | 102 | Frameshift | Y | N | N | N | N | N |
| 12 | c.449delA | 449 | p.Asn150fs | 150 | Deletion | N | N | N | Y | N | N |
| 13 | IVS2 + 1G>A | NA | NA | NA | Splice | N | N | N | N | N | N |
| 14 | IVS2 + 1G>A | NA | NA | NA | Splice | Y | N | N | Y | N | N |
| 15 | IVS2 + 1G>A | NA | NA | NA | Splice | Y | Y | Y | N | N | N |
| 16 | IVS2 + 1G>A | NA | NA | NA | Splice | N | N | N | N | N | N |
| 17 | c.238A>C | 238 | p.Ser80Arg | 80 | Missense | Y | N | N | N | N | N |
| 18 | c.238A>C | 238 | p.Ser80Arg | 80 | Missense | Y | N | N | Y | N | N |
| 19 | IVS1 + 7G>A | NA | NA | NA | Splice | Y | N | N | N | N | N |
| 20 | IVS1 + 7G>A | NA | NA | NA | Splice | Y | N | N | N | N | N |
| 21 | c.238A>C | 238 | p.Ser80Arg | 80 | Missense | Y | Y | N | N | N | N |
| 22 | c.238A>C | 238 | p.Ser80Arg | 80 | Missense | Y | Y | N | N | N | Y |
| 23 | c.238A>C | 238 | p.Ser80Arg | 80 | Missense | Y | N | N | Y | N | N |
| 24 | c.238A>C | 238 | p.Ser80Arg | 80 | Missense | Y | Y | N | Y | N | N |
| 25 | c.238A>C | 238 | p.Ser80Arg | 80 | Missense | N | N | N | Y | N | Y |
| 26 | c.238A>C | 238 | p.Ser80Arg | 80 | Missense | N | N | N | Y | N | N |
| 27 | c.238A>C | 238 | p.Ser80Arg | 80 | Missense | N | N | N | N | N | N |
| 28 | c.238A>C | 238 | p.Ser80Arg | 80 | Missense | N | N | N | N | N | N |
| 29 | c.499C>T | 499 | p.Arg167Trp | 167 | Missense | Y | N | N | Y | Y | N |
| 30 | c.499C>T | 499 | p.Arg167Trp | 167 | Missense | N | N | N | N | Y | N |
| 31 | Partial deletion | NA | NA | NA | Large deletion | N | N | N | Y | N | N |
| 32 | Partial deletion | NA | NA | NA | Large deletion | N | N | N | N | N | N |
| 33 | Partial deletion | NA | NA | NA | Large deletion | N | N | N | N | N | N |
| 34 | c.388G>T | 388 | p.Val130Phe | 130 | Missense | N | N | N | N | N | N |
| 35 | c.388G>T | 388 | p.Val130Phe | 130 | Missense | N | N | N | N | N | N |
| 36 | c.388G>T | 388 | p.Val130Phe | 130 | Missense | N | N | N | N | N | N |
| 37 | c.344delA | 344 | p.His115Profs*158 | NA | Frameshift | N | N | N | N | N | N |
| 38 | c.344delA | 344 | p.His115Profs*158 | NA | Frameshift | N | N | N | N | N | N |
| 39 | c.344delA | 344 | p.His115Profs*158 | NA | Frameshift | N | N | N | Y | N | N |
| 40 | c.344delA | 344 | p.His115Profs*158 | NA | Frameshift | Y | N | N | N | N | N |
| 41 | c.344delA | 344 | p.His115Profs*158 | NA | Frameshift | N | Y | N | N | N | N |
| 42 | c.344delA | 344 | p.His115Profs*158 | NA | Frameshift | N | N | N | N | N | N |
| 43 | c.320G>C | 320 | p.Arg107Pro | 107 | Missense | Y | Y | N | Y | Y | Y |
| 44 | c.320G>C | 320 | p.Arg107Pro | 107 | Missense | N | N | N | N | N | N |
| 45 | c.437delC | 437 | p.P146Lfs*13 | 146 | Frameshift | Y | N | N | Y | N | N |
| 46 | c.280G>T | 280 | p.Glu94Ter | 94 | Stop Gained | Y | N | N | Y | N | N |
| 47 | c.256C>T | 256 | p.Pro86Ser | 86 | Missense | N | N | N | Y | N | N |
| 48 | c.256C>T | 256 | p.Pro86Ser | 86 | Missense | Y | N | N | N | N | N |
| 49 | Partial deletion | NA | NA | NA | Large deletion | Y | N | N | N | N | N |
| 50 | Partial deletion | NA | NA | NA | Large deletion | Y | N | N | Y | N | N |
| 51 | c.371C>T | 371 | p.Thr124Ile | 124 | Missense | N | N | N | Y | Y | N |
| 52 | c.371C>T | 371 | p.Thr124Ile | 124 | Missense | N | N | N | Y | Y | N |
| 53 | c.302T>G | 302 | p.Leu101Arg | 101 | Missense | Y | N | N | N | N | N |
| 54 | c.499C>T | 499 | p.Arg167Trp | 167 | Missense | Y | N | Y | N | Y | N |
| 55 | c.500G>A | 500 | p.Arg167Gln | 167 | Missense | Y | N | N | N | Y | N |
| 56 | c.500G>A | 500 | p.Arg167Gln | 167 | Missense | N | N | N | N | Y | N |
| 57 | c.500G>A | 500 | p.Arg167Gln | 167 | Missense | Y | N | N | N | Y | N |
| 58 | c.500G>A | 500 | p.Arg167Gln | 167 | Missense | N | N | N | N | N | N |
| 59 | c.500G>A | 500 | p.Arg167Gln | 167 | Missense | N | N | N | N | N | N |
| 60 | c.453C>G | 453 | p.I151M | 151 | Missense | Y | N | N | Y | Y | N |
| 61 | c.453_454delCA | 453 | p.Leu153fs*20 | 153 | Frameshift | Y | N | N | Y | N | N |
| 62 | c.464-1G>C | 464 | NA | NA | Splice | Y | Y | N | Y | Y | N |
| 63 | c.481C>T | 481 | p.Arg161Ter | 161 | Nonsense | Y | N | N | Y | N | N |
| 64 | c.233A>G | 233 | p.Asn78Ser | 78 | Missense | Y | N | Y | N | N | N |
| 65 | c.233A>G | 233 | p.Asn78Ser | 78 | Missense | N | N | N | N | N | N |
| 66 | c.224_226delTCT | 224 | p.Phe76del | 76 | In-frame del | Y | N | N | N | N | N |
| 67 | c.224_226delTCT | 224 | p.Phe76del | 76 | In-frame del | N | N | N | N | N | N |
| 68 | Complete deletion | NA | NA | NA | Large deletion | Y | N | N | Y | N | N |
| 69 | c.337C>T | 337 | p.Arg113Ter | 113 | Nonsense | N | N | N | N | N | N |
| 70 | c.(?_ - 213)_(*3705_?)del | NA | NA | NA | Large deletion | Y | N | N | Y | N | N |
| 71 | c.(?_ - 213)_(*3705_?)del | NA | NA | NA | Large deletion | N | N | N | N | N | N |
| 72 | c.(?_ - 213)_(*3705_?)del | NA | NA | NA | Large deletion | Y | N | N | Y | N | N |
| 73 | c.(?_ - 213)_(*3705_?)del | NA | NA | NA | Large deletion | Y | Y | N | Y | Y | N |
| 74 | c.(?_ - 213)_(*3705_?)del | NA | NA | NA | Large deletion | N | N | N | N | N | N |
| 75 | c.(?_ - 213)_(*3705_?)del | NA | NA | NA | Large deletion | Y | Y | N | Y | N | N |
| 76 | c.(?_ - 213)_(*3705_?)del | NA | NA | NA | Large deletion | Y | N | N | Y | N | N |
| 77 | c.473T>C | 473 | p.Leu158Pro | 158 | Missense | Y | N | N | N | N | N |
| 78 | c.245G>C | 245 | p.Arg82Pro | 82 | Missense | Y | N | N | Y | N | N |
| 79 | c.245G>C | 245 | p.Arg82Pro | 82 | Missense | Y | N | N | Y | N | N |
| 80 | c.507delG | 507 | p.Met170TrpfsTer8 | 170 | Frameshift | N | N | N | N | N | N |
| 81 | c.1 - ?_340+?del | NA | NA | NA | Large deletion | Y | Y | N | N | N | N |
| 82 | c.1 - ?_340+?del | NA | NA | NA | Large deletion | Y | Y | Y | N | N | Y |
| 83 | c.388G>T | 388 | p.Val130Phe | 130 | Missense | Y | N | N | Y | N | Y |
| 84 | c.264G>T | 264 | p.Trp88Cys | 88 | Missense | N | Y | N | N | N | N |
| 85 | c.264G>T | 264 | p.Trp88Cys | 88 | Missense | N | Y | N | N | N | N |
| 86 | c.264G>T | 264 | p.Trp88Cys | 88 | Missense | N | N | N | N | N | N |
| 87 | c.264G>T | 264 | p.Trp88Cys | 88 | Missense | Y | N | N | Y | N | N |
| 88 | c.264G>T | 264 | p.Trp88Cys | 88 | Missense | N | N | N | N | N | N |
| 89 | c.496G>T | 496 | p.Val166Phe | 166 | Missense | Y | N | N | Y | Y | N |
| 90 | c.195G>C | 195 | p.Ser65= | 65 | Splice | Y | Y | N | Y | Y | N |
| 91 | c.371C>T | 371 | p.Thr124Ile | 124 | Missense | Y | Y | N | Y | N | Y |
| 92 | c.565delG | 565 | p.Glu189fs | 189 | Deletion | Y | Y | Y | Y | N | Y |
| 93 | Nt507C>A | 507 | Y98X | NA | UTR | Y | Y | Y | Y | N | N |
| 94 | c.388G>T | 388 | p.Val130Phe | 130 | Missense | N | Y | N | Y | Y | N |
| 95 | c.(?_ - 213)_(*3705_?)del | NA | NA | NA | Large deletion | Y | Y | Y | N | N | N |
| 96 | c.(?_ - 213)_(*3705_?)del | NA | NA | NA | Large deletion | Y | Y | N | N | N | N |
| 97 | c.388G>T | 388 | .Val130Phe | 130 | Missense | Y | N | N | N | N | N |

CNS – central nervous system, CCRCC – clear cell renal cell carcinoma, PNEN – pancreatic neuroendocrine neoplasm, RH – retinal hemangioblastoma, PPGL – pheochromocytoma/paraganglioma, EPI – epididymal tumor.

Mutations are described using 'p.' when referring to the VHL protein sequence, and 'c.' for the VHL cDNA sequence. Amino acids are in single-letter notation. Mutations are reported in accordance with the nomenclature for the description of sequence variations as proposed by the Human Genome Variation Society (www.hgvs.org/mutnomen/).

Figure S1 – Categorization of families according to phenotype.


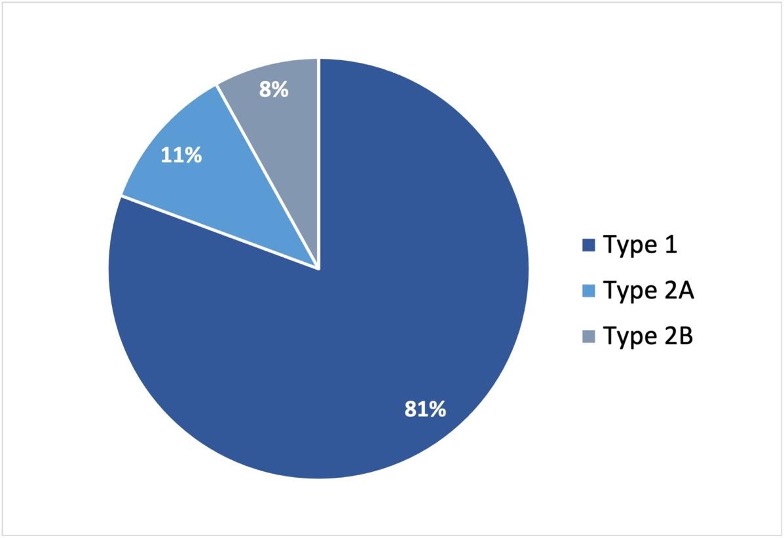


Figure S2 - Treatment modalities reported by participants with VHL manifestations.


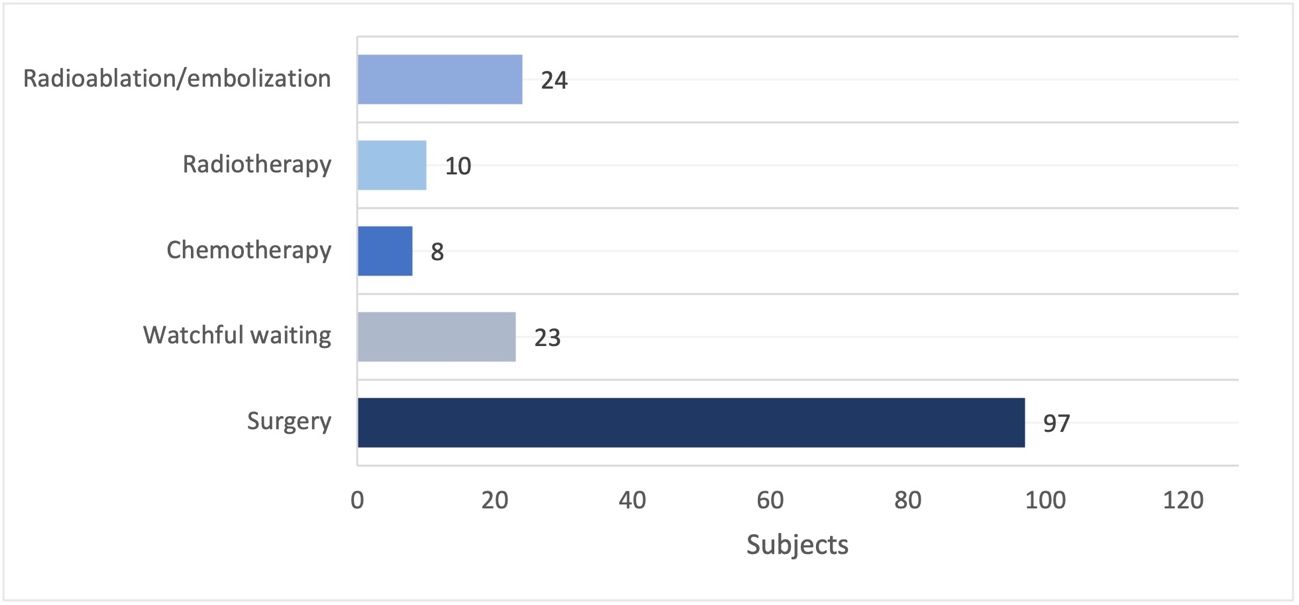


Figure S3 - Reference medical specialty according to the participants.


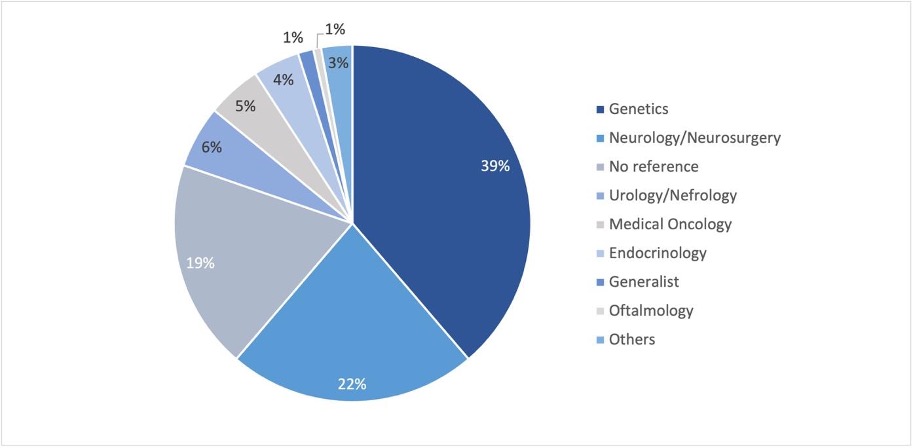


Figure S4 - Frequency of follow-up tests reported by the 115 participants in medical follow-up


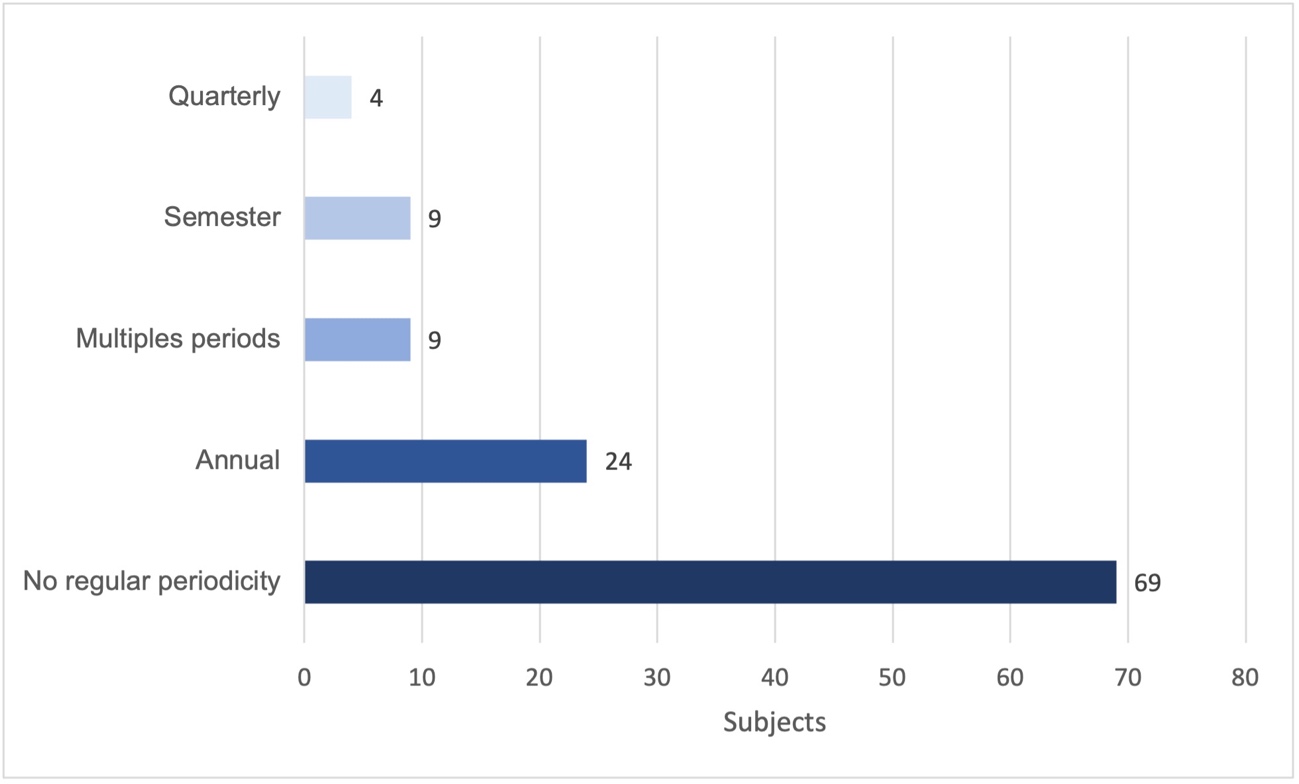


Figure S5 - Regular follow-up of VHL-related target lesions (91 participants)


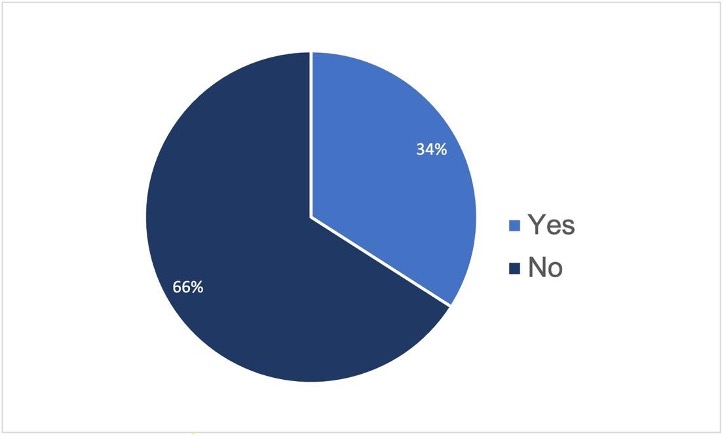

Supplement: Supplementary file 1 — Appendix S1 [file MGG3-11-e2136-s001.docx]
